# Inulin-type fructan improves diabetic phenotype and gut microbiota profiles in rats

**DOI:** 10.7717/peerj.4446

**Published:** 2018-03-01

**Authors:** Qian Zhang, Hongyue Yu, Xinhua Xiao, Ling Hu, Fengjiao Xin, Xiaobing Yu

**Affiliations:** 1 Key Laboratory of Endocrinology, Translational Medicine Center, Ministry of Health, Department of Endocrinology, Peking Union Medical College Hospital, Peking Union Medical College, Chinese Academy of Medical Sciences, Beijing, China; 2 Department of Endocrinology, Shanxi Provincial People’s Hospital, Taiyuan, China; 3 Institute of Food Science and Technology, Chinese Academy of Agricultural Sciences (CAAS), Beijing, China; 4 Fengning Ping’an High-tech Industrial Co., Ltd., Hebei Province, China

**Keywords:** Gut microbiota, Inulin, Soluble fiber, Diabetes

## Abstract

**Background & Aims:**

Accumulating research has addressed the linkage between the changes to gut microbiota structure and type 2 diabetes (T2D). Inulin is one type of soluble dietary fiber that can alleviate T2D. As a prebiotic, inulin cannot be digested by humans, but rather is digested by probiotics. However, whether inulin treatment can benefit the entire gut bacteria community remains unknown. In this study, we evaluated the differences in gut microbiota composition among diabetic, inulin-treated diabetic, normal control, and inulin-treated normal control rats.

**Methods:**

A diabetic rat model was generated by a high-fat diet and streptozotocin injections (HF/STZ). Inulin was orally administered to normal and diabetic rats. To determine the composition of the gut microbiota, fecal DNA extraction and 16S rRNA gene 454 pyrosequencing were performed.

**Results:**

We found that inulin treatment reduced fasting blood glucose levels and alleviated glucose intolerance and blood lipid panels in diabetic rats. Additionally, inulin treatment increased the serum glucagon-like peptide-1 (GLP-1) level, reduced serum IL-6 level, *Il6* expression in epididymal adipose tissue, and *Pepck*, *G6pc* expression in liver of diabetic rats. Pyrophosphate sequencing of the 16s V3–V4 region demonstrated an elevated proportion of *Firmicutes* and a reduced abundance of *Bacteroidetes* at the phylogenetic level in diabetic rats compared to normal control rats. The characteristics of the gut microbiota in control and inulin-treated rats were similar. Inulin treatment can normalize the composition of the gut microbiota in diabetic rats. At the family and genus levels, probiotic bacteria *Lactobacillus* and short-chain fatty acid (SCFA)-producing bacteria *Lachnospiraceae*, *Phascolarctobacterium*, and *Bacteroides* were found to be significantly more abundant in the inulin-treated diabetic group than in the non-treated diabetic group. In addition, inulin-treated rats had a lower abundance of *Desulfovibrio*, which produce lipopolysaccharide (LPS). The abundance of *Lachnospiraceae* was negatively correlated with the blood glucose response after a glucose load.

**Conclusion:**

In summary, diabetic rats have different gut microbiota from control rats. Inulin treatment can alleviate gut microbiota dysbiosis in T2D model rats. Moreover, inulin treatment enhanced serum GLP-1 level to suppress IL-6 secretion and production and hepatic gluconeogenesis, resulted in moderation of insulin tolerance.

## Introduction

The prevalence of type 2 diabetes (T2D) is dramatically increasing worldwide. T2D is characterized by low-grade inflammation, insulin resistance, and pancreatic β-cell failure. Recently, increasing evidence has addressed the link between T2D and gut microbiota. The underlying mechanism of T2D is also of interest. In addition to genetic, physiological and environmental factors, gut microbiota also markedly contribute to the incidence of T2D (Baothman et al., 2016; Tai, Wong & Wen, 2015). T2D patients have a moderate degree of gut microbial dysbiosis (Qin et al., 2012). Germ-free mice exhibit reduced susceptibility to high-fat diet-induced obesity, insulin resistance, and glucose intolerance (Rabot et al., 2010). Moreover, transplantation of gut microbiota from ob/ob mice to germ-free mice leads to obesity and insulin resistance (Turnbaugh et al., 2006). These studies suggest that changes in gut microbiota may be potential targets for the treatment of T2D.

As a prebiotic, inulin (extracted from chicory root), cannot be hydrolyzed by human small intestinal digestive enzymes but is fermented by certain bacteria, such as *Bifidobacterium* and *Lactobacillus*, in the large intestine with lactate and short-chain fatty acids (SCFAs) (Alles et al., 1996). Inulin oligofructose (OFS, a chicory inulin-type fructan with a low degree of polymerization) has been shown to reduce blood glucose and moderate insulin resistance in diabetic rats and mice (Busserolles et al., 2003; Cani et al., 2005a; Cani et al., 2006b). In healthy humans, OFS promotes satiety after meals (Cani et al., 2005a; Cani et al., 2006a). The useful effects of inulin are primarily related to an increase in glucagon-like peptide-1 (GLP-1) (Cani et al., 2005b). GLP-1 is released from intestinal L cells upon stimulation by nutrients. GLP-1 promotes insulin secretion and pancreas β-cell proliferation, controls glycogen synthesis in muscle cells, and enhances satiety (Meier & Nauck, 2005).

However, the effect of inulin on the gut microbiome in diabetic rats is still not clear. Recently, 16S rRNA gene sequencing in gut microbiota was utilized to simultaneously assess hundreds of gut bacteria. By utilizing 16s rRNA gene sequencing, we identified the changes that occur in the gut microbiota of inulin-treated diabetic rats. We hypothesized that inulin treatment would normalize gut microbiota in diabetic rats. The purpose of this research was to identify the beneficial bacteria in the gut microbiota that are responsible for the effects of inulin treatment.

## Materials and Methods

### Study design and animal experiments

All procedures were undertaken with the approval of the Animal Care Committee of the Peking Union Medical Hospital Animal Ethics Committee (Project XHDW-2015-0051, 15 Feb 2015), and all efforts were made to minimize suffering. Five-week-old male Sprague-Dawley (SD) rats (158.3 ± 14.8 g) were obtained from the Institute of Laboratory Animal Science, Chinese Academy of Medical Sciences and Peking Union Medical College (Beijing, China, SCXK-2014-0013). All rats were maintained in cages at 24 ± 1 °C with lights on from 6:00 a.m. to 6:00 p.m. and were given free access to food and water.

Control rats were fed a standardized diet (kcal %: 10% fat, 20% protein, and 70% carbohydrate; 3.85 kcal/gm), and experimental rats were fed a high-fat diet (kcal %: 45% fat, 20% protein, and 35% carbohydrate; 4.73 kcal/gm; Research Diet, New Brunswick, NJ, USA) for four weeks and then intraperitoneally injected with streptozotocin (STZ, 30 mg/kg body weight) to induce diabetes (Chen et al., 2017). The diabetic animals were fed continuously on the high-fat diet throughout the remainder of the study. Fasting blood glucose (FBG) >11.1 mmol/L was determined to be the standard concentration for the type 2 diabetes model. Diabetic rats were randomly divided into two groups: an inulin-treated diabetic group (DM + inulin, Vilof™ Soluble Dietary Fiber; BAHEAL Medical Inc., Qingdao, China and Fengning Ping’an High-tech Industrial Co., Ltd., Hebei, China, orally administered 3 g Vilof™ Soluble Dietary Fiber powder/kg body weight/day, *n* = 6) and an untreated-diabetic group (DM, orally administered the same volume of normal saline, *n* = 6). Vilof™ Soluble Dietary Fiber powder contains 91% inulin-type fructan and 9% sucrose, fructose, and glucose. Control rats were randomly divided into an untreated control group (CON, *n* = 6, administered normal saline) and an inulin-treated control group (CON + inulin, *n* = 6, administered 3 g Vilof™ Soluble Dietary Fiber powder/kg body weight/day). After 12 weeks, fresh stool samples were obtained by stimulating the anus and were immediately frozen and stored at −80 °C for subsequent analysis. After 12 h of food deprivation, the rats were anesthetized (ketamine 100 mg/kg i.p.; Pharmacia and Upjohn Ltd., Crawley, UK), and blood samples were collected from the intraorbital retrobulbar plexus at 8 a.m. (Chen et al., 2017). The rats were thensacrificed. Epididymal fat and liver was quickly collected and kept at −80 °C.

### Measurements of body weight and fasting blood glucose (FBG)

Body weight and FBG were monitored monthly with Bayer Contour TS glucometer (Bayer, Hamburg, Germany).

### Oral glucose tolerance test (OGTT) and area under the curve

After fasting for 12 h, an OGTT was performed. Glucose (2 g/kg body weight) was orally administered to the rats. Glucose levels in the blood obtained from the tail were recorded before and 30, 60, and 120 min after the glucose load. The area under the curve (AUC) was calculated by the linear trapezoid method (Zhang et al., 2016).

### Measurement of serum insulin, GLP-1, lipid profile, interleukin 6 (IL-6), and HOMA-IR

After 12 h of food deprivation, rat serum was obtained to analyze insulin (ELISA, Millipore, Billerica, MA, USA), GLP-1 (ELISA, Millipore, Billerica, MA, USA), total cholesterol (TC, enzyme end-point method; Roche Diagnostics, GmbH, Mannheim, Germany), triglyceride (TG, enzyme end-point method, Roche Diagnostics, GmbH, Mannheim, Germany), and IL-6 (ELISA, Millipore, Billerica, MA, USA) concentrations, according to the manufacturer’s instruction. The homeostasis model assessment of insulin resistance (HOMA-IR) was calculated by the following formula: fasting blood glucose (FBG, mmol/L) × fasting serum insulin (µIU/mL)/ 22.5.

### Measurement of liver TG content

Liver tissue (100 mg) was homogenized in 350 µL ethanolic KOH, then incubated overnight at 55 °C. The next day, 1,000 µL H2O: EtOH (1:1) was added to the tube. The samples were centrifuged for 5 min. The supernatant was used to measure the content of TG using a colorimetric kit (Biovision, Milpitas, CA, USA).

### Quantitative PCR assay of *Il6* mRNA expression in adipose tissue, *G6pc* and *Pepck* mRNA expression in liver

Total RNA from epididymal fat and liver was extracted by using RNA Isolation Kit (RNeasy kit, Qiagen, Valencia, CA, USA) according to the manufacturer’s instructions. RNA was reverse transcribed by Superscript II (Invitrogen, Carlsbad, CA, USA). Quantitative PCR was performed by using SYBR Green Master Mix (Applied Biosystems, Foster City, CA, USA). The primer was produced by Applied Biosystems (Foster City, CA, USA). Specific primers for rat *Il6* mRNA were 5′-CTGGTCTTCTGGAGTTCCGT-3′ (forward) and 5′-TGGTCCTTAGCCACTCCTTCT-3′ (reverse), rat *Pepck* mRNA were 5′-CCAAGAGCAGAGAGACACCG-3′ (forward) and 5′-ATACATGGTGCGGCCTTTCA-3′ (reverse), rat *G6pc* mRNA were 5′-GCGTGCCATAGGACTCATCA-3′ (forward) and 5′-CACCAGCAAACAATTGCCCA-3′ (reverse). The products were run in the ABI Prism 7700 Sequence Detection system (Applied Biosystems, Foster City, CA, USA). The cycling conditions were set at 95 °C for 15 min, followed by 40 cycles of 95 °C for 15 s, 55 °C for 1 min and 72 °C for 1 min. *Gadph* was used for normalization. Relative quantification of mRNA was calculated using the comparative threshold cycle (Ct) method.

### Fecal DNA extraction and amplification

Total DNA from fecal samples was isolated using a QIAmp DNA Stool Mini Kit (Qiagen, Valencia, CA, USA) according to the manufacturer’s instructions. The 16S rRNA hypervariable regions V3–V4 were PCR amplified using barcoded, gene specific primers 341F (5′-CCTAYGGGRBGCASCAG-3′) and 806R (5′-GGACTACNNGGGTATCTAAT-3′) with Phusion High-Fidelity PCR Master Mix (New England Biolabs, Ipswich, MA, USA) followed by library preparation (TruSeq DNA PCR-Free Sample Preparation Kit; Illumina, San Diego, CA, USA).

### Pyrophosphate sequencing and primary data analysis

Pyrosequencing was performed on an Illumina HiSeq 2500 platform (San Diego, CA, USA), and 250-bp paired-end reads were generated. Paired-end reads were connected using FLASH (John Hopkins University School of Medicine, Baltimore, MD, USA) (Magoc & Salzberg, 2011). Raw reads were filtered with a specific standard to gain high quality reads using QIIME version 1.7.0 (Caporaso et al., 2010). Sequences analysis was performed using UPARSE version 7.0.1001 (Edgar, 2013). The operational taxonomic units (OTUs) were generated from ≥97% similarity sequences. Representative sequences for each OTU were screened for further annotation.

### Taxon composition and species diversity analysis

OTUs were annotated with taxonomic information based on the RDP classifier version 2.2 (Wang et al., 2007) algorithm using the Greengene database (DeSantis et al., 2006). OTU abundance data were normalized using a standard sequence number corresponding to the sample with least sequences. The relative proportion of each OTU was examined at the Phylum, Class, Order, Family, Genus and Species levels.

Alpha (within a community) and beta (between communities) diversity were analyzed with QIIME version 1.7.0. For alpha diversity, Chao1 and Shannon indices were calculated to identify community richness and diversity, respectively. For beta diversity, principal coordinates analysis (PCoA) plots were generated using both weighted (which considers the abundance of each species) and unweighted (which considers the presence or absence of each species) UniFrac (Lozupone & Knight, 2005). Unweighted pair-group method with arithmetic means (UPGMA) clustering was employed as the hierarchical clustering method to interpret the distance matrix using average linkage. In addition, linear discriminant analysis of the effect size (LEfSe) was performed to calculate OTU abundance and to determine the differences among groups (Segata et al., 2011). The threshold for the logarithmic linear discriminant analysis (LDA) score was >3.0.

### Data analysis

The data are expressed as the mean ± SD. When the data were normal and variances were equal, differences among the groups were analyzed using one-way ANOVA followed by Tukey’s post hoc test. Otherwise, the Kruskal–Wallis test and the Mann–Whitney test were applied. Spearman’s correlation analysis was performed to identify the correlations. A *P*-value ≤0.05 was considered statistically significant (Prism version 5.0 for Windows; GraphPad Software, San Diego, CA, USA).

## Results

### Body weight

**Figure 1:**
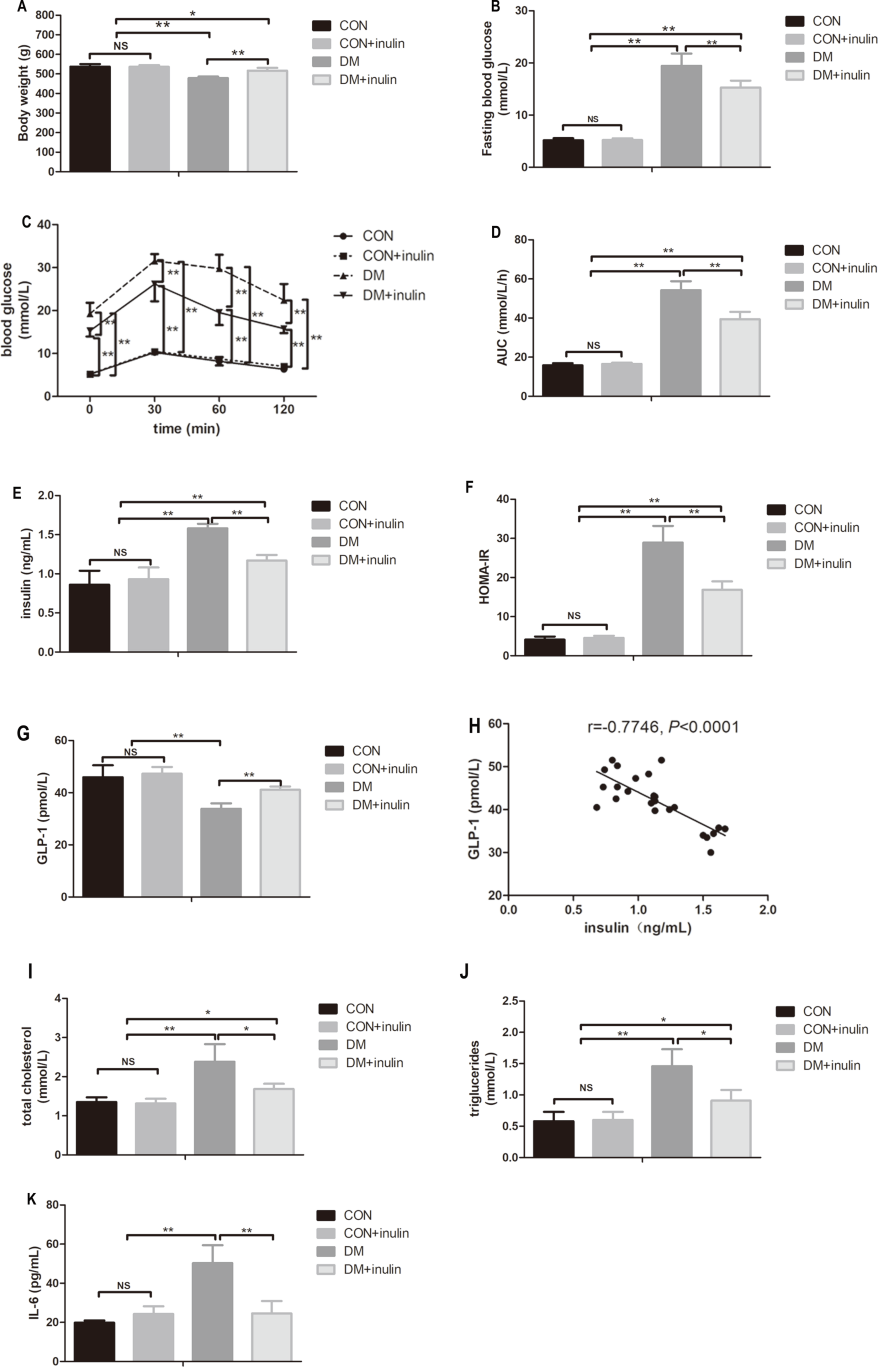
The effect of inulin on body weight, fasting blood glucose, blood glucose in OGTT, serum insulin, HOMA-IR index, GLP-1, blood lipid panels, pro-inflammation cytokine, and hepatic TG content. (A) Body weight, (B) fasting blood glucose, (C) blood glucose in oral glucose tolerance test, (D) area under curve (AUC) in OGTT, (E) serum insulin, (F) HOMA-IR, (G) GLP-1, (H) total cholesterol (TC), (I) triglyceride (TG), (J) interleukin 6 (IL-6), and (K) hepatic TG content. Data are presented as means ± SD (*n* = 6). Data was *P < 0.05, **P < 0.01, ^NS^ not significant (one-way ANOVA followed by Tukey’s post hoc test).

Diabetic rats exhibited significantly lower body weight (*P* < 0.01, Fig. 1A), while inulin-treated diabetic rats had an increased body weight compared with the control diabetic rats (*P* < 0.01, Fig. 1A).

### Fasting blood glucose level and glucose tolerance

Diabetic rats showed statistically elevated FBG levels compared to normal control rats (*P* < 0.01, Fig. 1B), and inulin treatment reduced FBG levels in diabetic rats (*P* < 0.01, Fig. 1B). According to the oral glucose tolerance test, blood glucose levels significantly increased before and after glucose load (*P* < 0.01, Fig. 1C) in diabetic rats. The elevated glucose area under the curve (AUC) on the OGTT (*P* < 0.01) in diabetic rats indicated severe glucose intolerance in diabetic rats (Fig. 1D). Inulin treatment significantly reduced blood glucose levels before and after glucose load (*P* < 0.05 or *P* < 0.01) in diabetic rats (Fig. 1C). Similarly, diabetic rats treated with inulin treatment exhibited a reduced glucose AUC by 25.8% (*P* < 0.01, Fig. 1D).

### Fasting insulin and homeostasis model assessment of insulin resistance (HOMA-IR)

Dramatic increases in serum insulin levels and HOMA-IR in diabetic rats were noted (*P* < 0.01, Figs. 1E and 1F). Inulin treatment reduced serum insulin levels and HOMA-IR in diabetic rats (*P* < 0.01, Figs. 1E and 1F).

### Serum biochemical parameters

Fasting serum GLP-1 levels decreased in the diabetic group (*P* < 0.01), while inulin treatment increased fasting serum GLP-1 levels (*P* < 0.01, Fig. 1G). Serum TC, TG, and IL-6 significantly increased in the DM group (*P* < 0.01, Figs. 1H–1J). Inulin treatment reduced serum TC, TG and IL-6 levels in diabetic rats (*P* < 0.05, Figs. 1H–1J).

### Liver TG content

Liver TG content in diabetic rats significantly increased (*P* < 0.01, Fig. 1K). Inulin treatment reduced liver TG content slightly (*P* > 0.05, Fig. 1K).

### Epididymal fat *Il6* expression and hepatic *Pepck* and *G6pc* expression

**Figure 2:**
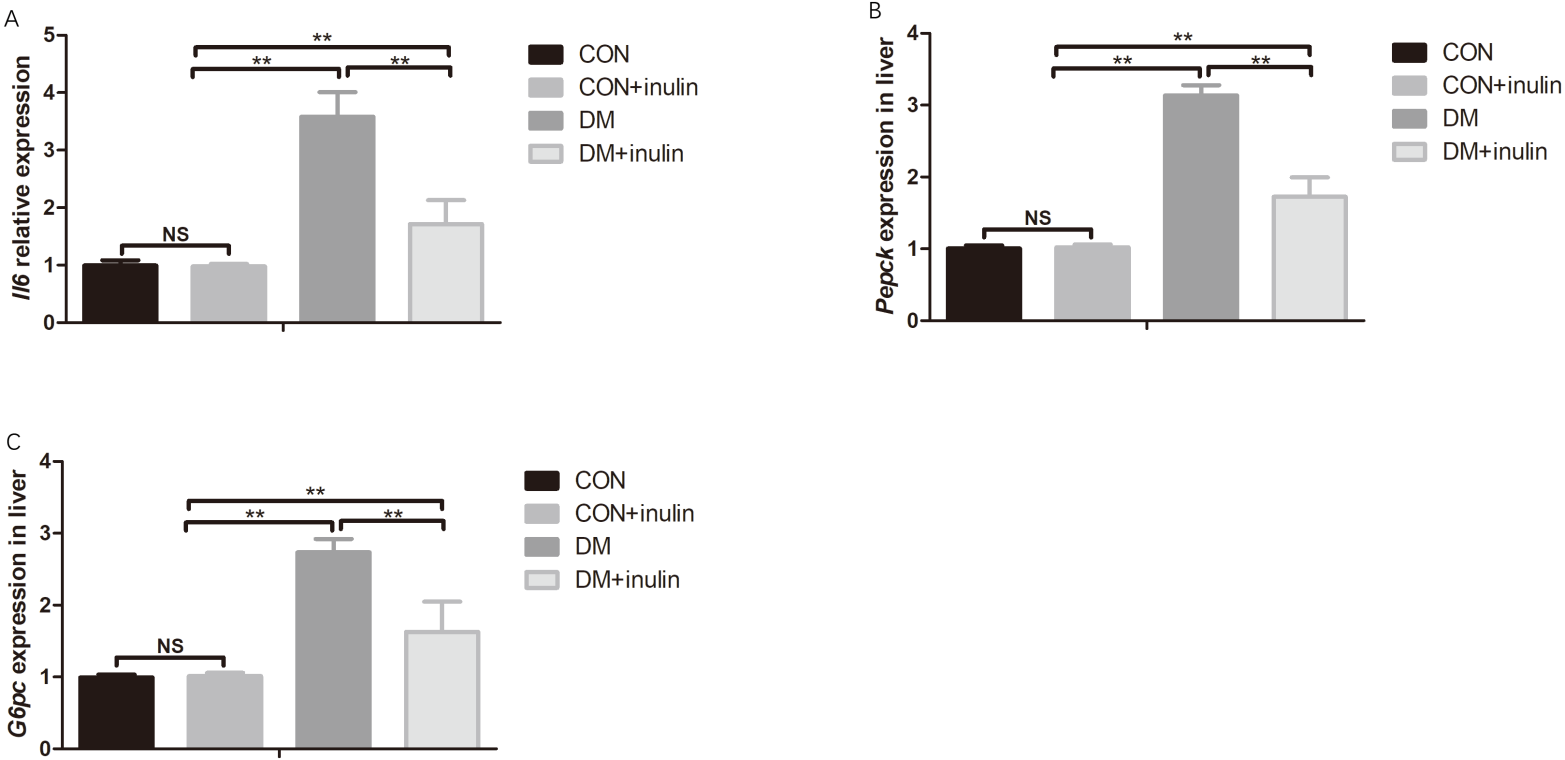
Pro-inflammatory cytokine and gluconeogenesis marker expression in white adipose tissue and liver. (A) *Il6* expression in epididymal fat, (B) *Pepck* expression in liver, and (C) *G6pc* expression in liver. **P < 0.01, ^NS^ not significant (one-way ANOVA followed by Tukey’s post hoc test).

The expression of *Il6* in white adipose tissue and hepatic gluconeogenesis markers (*Pepck* and *G6pc*) expression increased significantly in diabetic rats (*P* < 0.01, Figs. 2A–2C). Inulin treatment reduced epididymal fat *Il6* expression and hepatic *Pepck* and *G6pc* expression (*P* < 0.01, Figs. 2A–2C).

### Characterization of gut microbiota

To describe the composition of the gut microbiota, a total of 1,402,994 pyrosequencing reads (69,281 unique sequences) were obtained from 24 stool samples. The 16s sequence data generated in this study were submitted to the NCBI Sequence Read Archive (SRA) database (accession number SRP095682). Among them, 1,108,097 reads (48,153 sequences) were generated after quality filtering, with an average of 2,386 ± 1,699 sequences being recovered per sample. The high-quality sequences were then delineated into 873 operational taxonomic units (OTUs) at the similarity cutoff of 97%.

**Figure 3:**
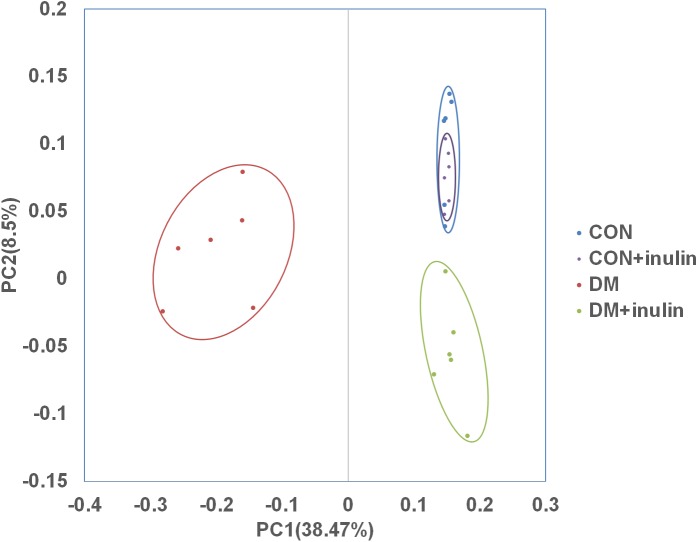
PCoA of unweighted Unifrac distances of the gut bacterial communities. Pyrosequencing data was analyzed with QIIME and subjected to unweighted Unifrac analysis. Component 1 explains 38.47% of the variation, component 2 explains 8.5% of the variation.

In normal rats, inulin treatment did not affect OTU number, the Shannon diversity or the Chao1 index (Table 1). However, in diabetic rats, inulin treatment significantly reduced the Shannon diversity index of the gut microbiota (*P* < 0.05, Table 1). The richness of the gut microbiota was also significantly reduced by inulin treatment in diabetic rats, as shown by the Chao1 index (*P* < 0.01, Table 1). To compare the beta diversity of gut microbiota among groups, principal coordinate analysis (PCoA) was carried out. As shown in Fig. 3, the first two principal coordinates of PCoA (components 1 and 2) were separated into CON, DM, and DM + inulin groups. However, the CON and CON + inulin groups shared some overlapping regions.

**Table 1:** Alpha diversity indices.

	CON	CON + inulin	DM	DM + inulin
OTUs	561.8 ± 27.6	556.8 ± 22.8	499.5 ± 29.9^**^^,^^##^	423.5 ± 48.5^**^^,^^##^^,^^$$^
Chao1	608.3 ± 23.7	588.0 ± 33.1	556.0 ± 32.2^**^	467.3 ± 55.5^**^^,^^##^^,^^$$^
Shannon	6.85 ± 0.29	6.77 ± 0.26	6.39 ± 0.34^*^^,^^#^	5.93 ± 0.40^**^^,^^##^^,^^$^

**Notes.**

Data are presented as means ± SD (*n* = 6). ^∗^*P* < 0.05, ^∗∗^*P* < 0.01 versus CON, ^#^*P* < 0.05, ^##^*P* < 0.01 versus CON +inulin; ^$^*P* < 0.05, ^$$^*P* < 0.01 versus DM (one-way ANOVA followed by Tukey’s post hoc test).

### Relative abundances of different bacteria in the gut microbiota after inulin treatment

**Figure 4:**
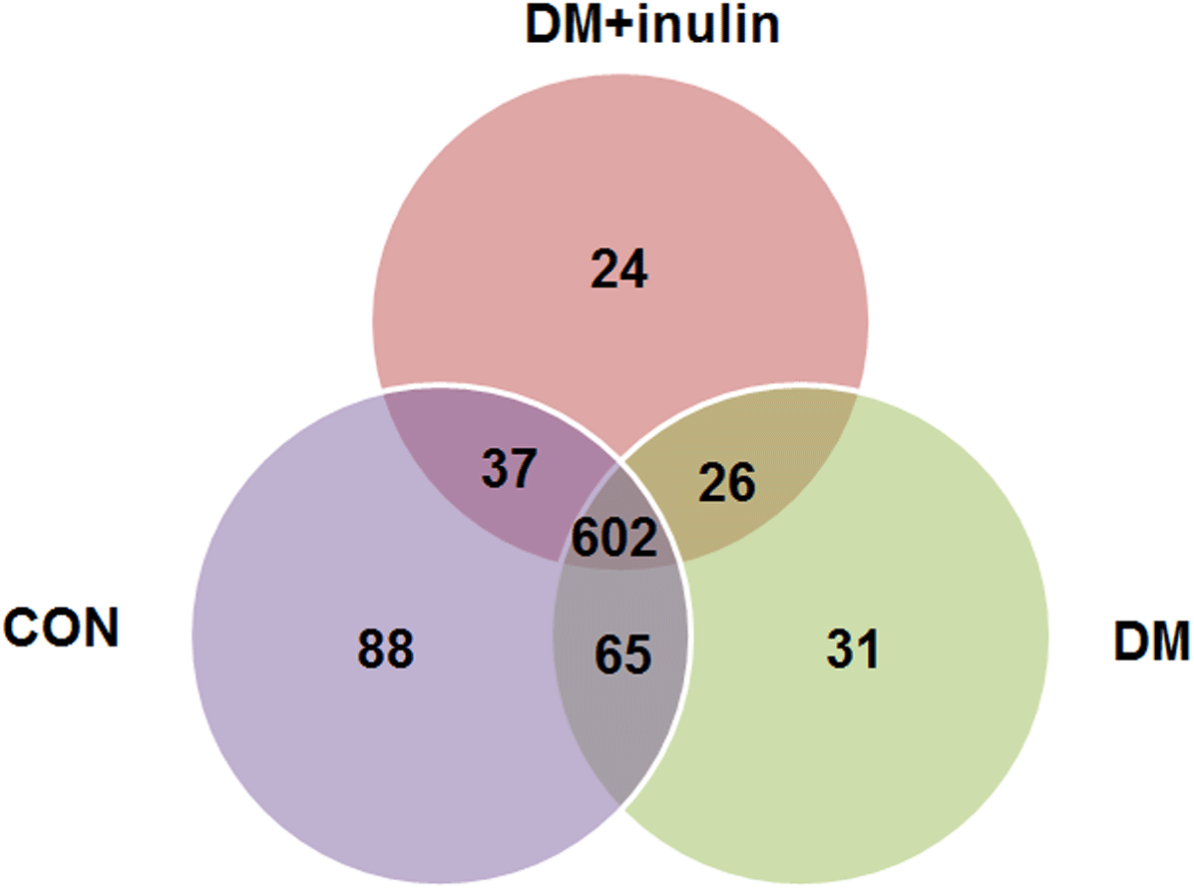
Shared OUT analysis of the different groups. Venn diagram showing the unique and shared OTUs (3% distance level) in the different groups.

As shown above, the CON and CON + inulin groups had similar alpha and beta diversities in gut microbiota. Next, we explored the similarities and differences of species distribution in the CON, DM, and DM + inulin groups. As shown in Fig. 4, we found that there were 602 species shared among the three groups, accounting for approximately three or four of the OTUs in each group. Of note, 88 species were found in the CON group, 31 OTUs in the DM group, and 24 OTUs in the DM + inulin group.

**Figure 5:**
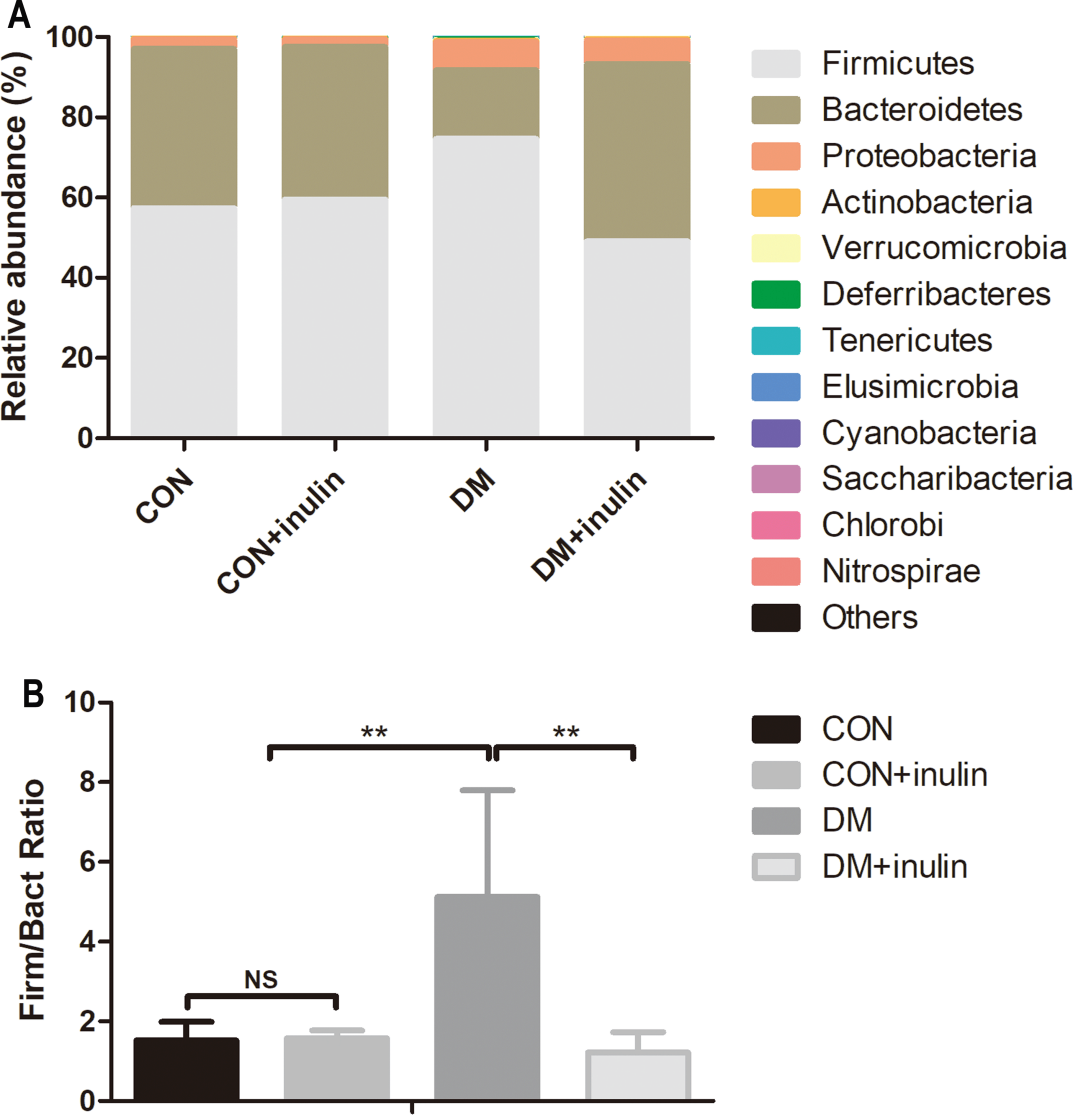
Fecal microbial communities at the phyla level. (A) Relative abundance of gut microbiota at the phyla level, and (B) the ratio of *Bacteroidetes*: *Firmicutes*. Data are presented as means ± SD (*n* = 6). **P < 0.01, ^NS^ not significant (one-way ANOVA followed by Tukey’s post hoc test).

Consistent with other reports, the primary phyla were *Bacteroidetes* and *Firmicutes* in all samples. The DM group had an elevated relative abundance of *Firmicutes* (*P* < 0.01) and a reduced abundance of *Bacteroidetes* (*P* < 0.01). Inulin treatment reduced the relative abundance of *Firmicutes* (*P* < 0.01) and increased the abundance of *Bacteroidetes* (*P* < 0.01, Fig. 5A) in diabetic rats. The ratio of *Firmicutes*-*Bacteroidetes* has been suggested to be an indicator of gut microbial imbalance related to high-fat diet (Hildebrandt et al., 2009; Murphy et al., 2010; Turnbaugh et al., 2006). Our results showed that DM rats had a higher ratio of *Firmicutes*-*Bacteroidetes* (*P* < 0.01), whereas inulin treatment reuced this ratio (*P* < 0.01, Fig. 5B). Moreover, inulin treatment reduced the relative abundance of *Tenericutes* in diabetic rats (*P* < 0.05, Fig. 5A).

**Figure 6:**
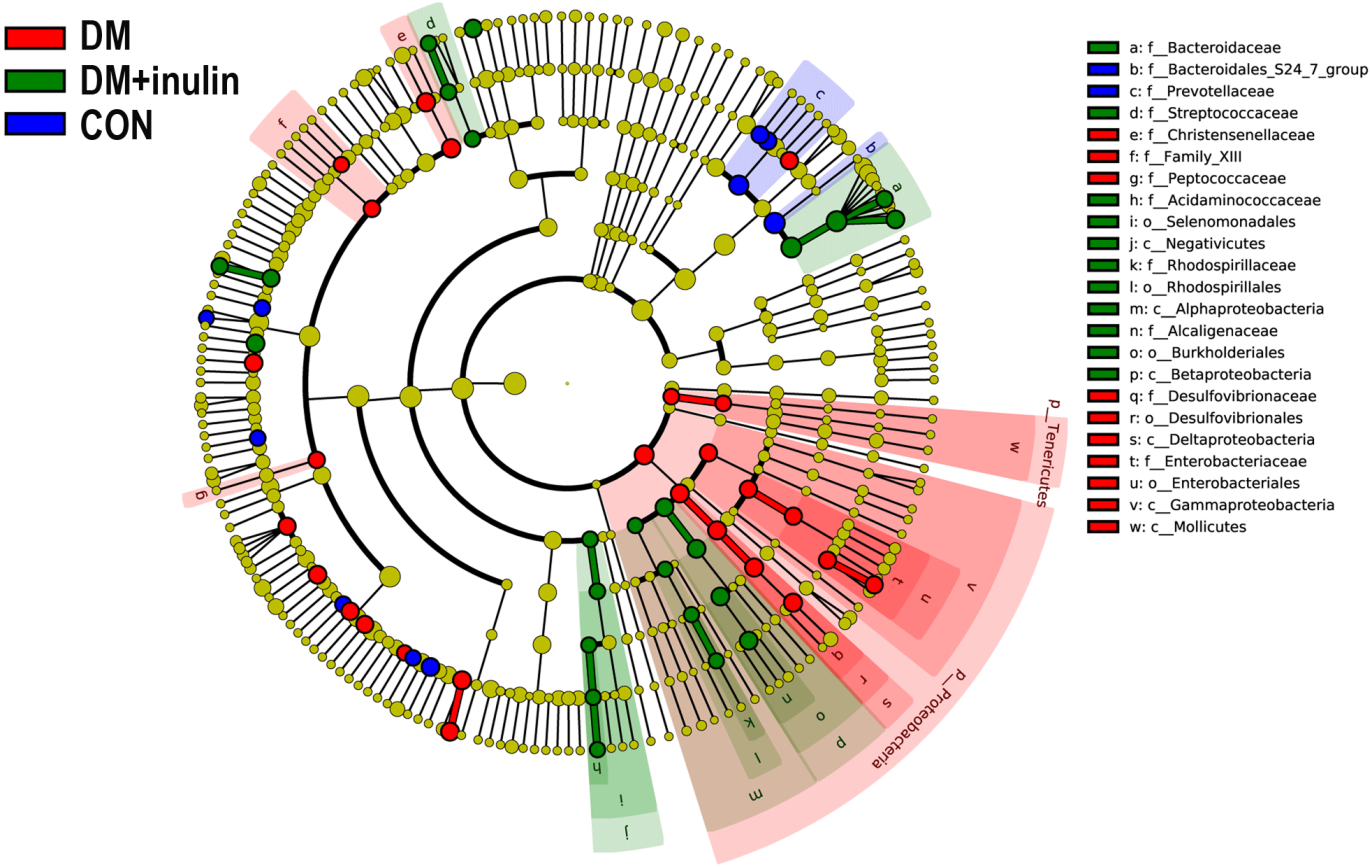
Cladogram indicating statistical differences of microbial populations in rats.

**Figure 7:**
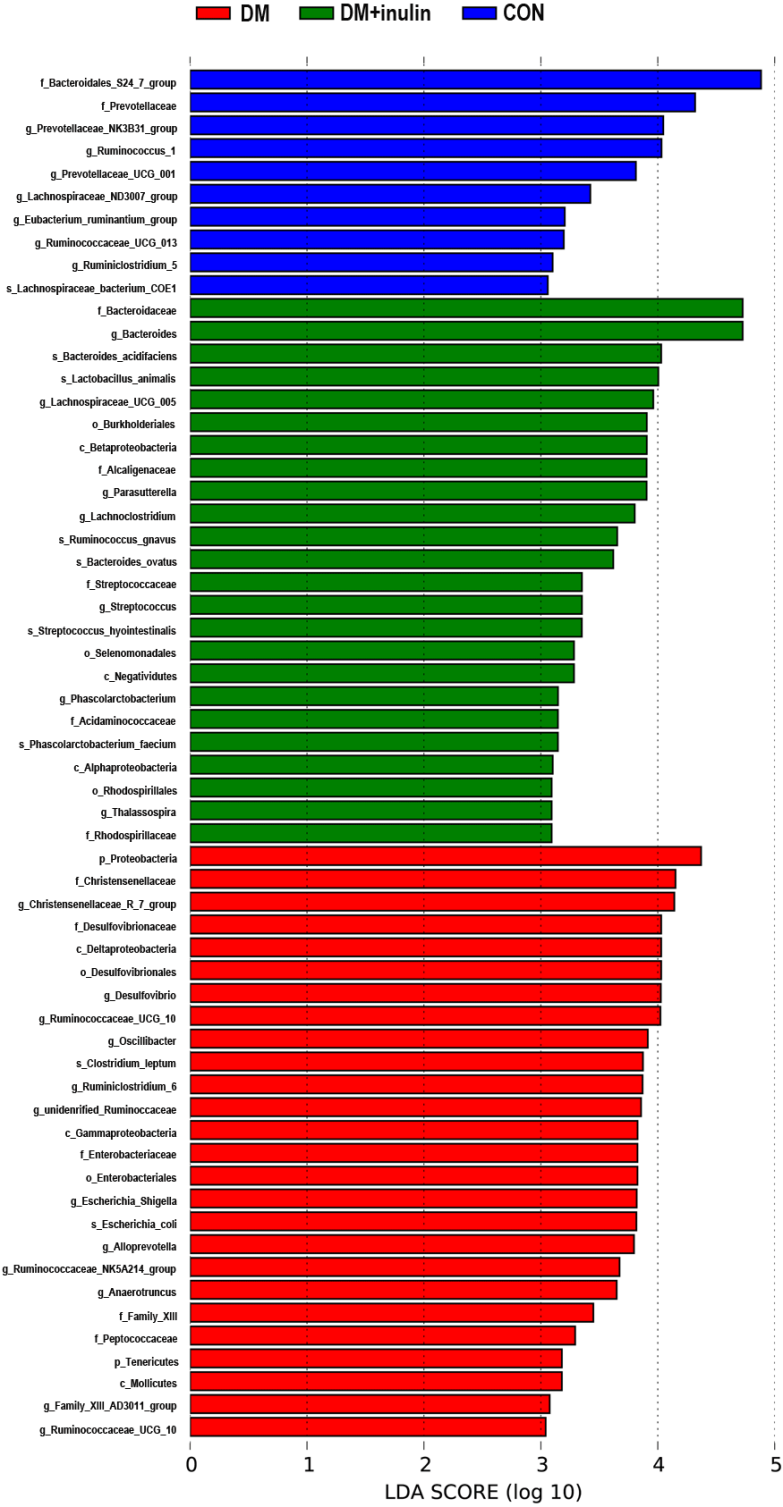
LDA scores indicating statistical differences of microbial populations in rats.

According to our LDA of the effective size, the abundances of many taxa were significantly different among the CON, DM, and DM + inulin groups (Figs. 6 and 7). At the family level, the *Streptococcaceae* and *Acidaminococcaceae* abundances were elevated in the DM + inulin group compared to the DM group, whereas the *Christensenellaceae*, *Peptococcaceae*, and *Desulfovibrionaceae* abundances were reduced in the DM + inulin group (*P* < 0.05, Table 2). At the genus level, the *Phascolarctobacterium*, *Streptococcus*, *Lachnoclostridium*, and *Parasutterella* abundances were all significantly elevated by inulin treatment, whereas the *Ruminococcaceae_NK4A214_group*, *Alloprevotella*, *Ruminococcaceae_UCG_010*, *Ruminiclostridium_6*, *Christensenellaceae_R_7_group*, *Desulfovibrio*, and *Oscillibacter* abundances were reduced by inulin treatment in diabetic rats (*P* < 0.05, Table 2). At the species level, *Lactobacillus_animalis, Ruminococcus_gnavus, Phascolarctobacterium_faecium, Streptococcus_hyointestinalis,* and *Bacteroides_acidifaciens* were more abundant in the DM + inulin group (*P* < 0.05, Table 2).

**Table 2:** The relative abundance (%) of bacterial groups that showed statistical significance based on the LEfSe method.

Bacterial group	*P*-value		Percentual abundance (%)		
	LDA	Kruskal–Wallis	CON	DM	DM + inulin
*Streptococcaceae*	3.351	0.013	0.135(0.013–0.920)	0.042(0.037–0.080)	0.227(0.150–2.070)^##^
*Escherichia_Shigella*	3.819	0.044	0.168(0.043–0.313)	0.238(0.060–4.940)	1.313(0.160–3.003)^*^
*Phascolarctobacterium*	3.146	0.027	0.000(0.000–0.037)	0.000(0.000–0.017)	0.030(0.000–1.027)^#^
*Bacteroides*	4.726	0.006	3.168(0.880–17.521)	7.917(3.180–14.854)	12.584(9.930–28.454)^**^
*Ruminococcus_1*	4.032	0.001	2.422(0.680–4.080)	1.538(0.703–1.860)	0.263(0.067–1.103)^***^
*Enterobacteriales*	3.826	0.016	0.187(0.043–0.353)	0.273(0.070–5.040)	1.357(0.263–3.087)^*^
*Bacteroides_ovatus*	3.619	0.023	0.062(0.013–0.297)	0.107(0.013–0.190)	0.447(0.053–3.743)^*^
*Desulfovibrionales*	4.029	0.013	1.065(0.517–1.923)	2.433(1.827–5.147)^*^	0.853(0.557–5.707) ^#^
*unidentified_Ruminococcaceae*	3.857	0.009	1.505(0.457–2.087)	2.650(1.677–5.050)^**^	1.663(0.440–4.993)
*_Eubacterium__ruminantium_group*	3.205	0.045	0.082(0.003–2.277)	0.028(0.007–0.087)	0.007(0.000–0.220)^*^
*Ruminococcaceae_NK4A214_group*	3.673	0.012	0.957(0.577–2.143)	1.273(0.500–2.513)	0.507(0.213–1.000)^#^
*Prevotellaceae_UCG_001*	3.812	0.001	0.950(0.360–3.397)	0.077(0.007–0.153)^**^	0.073(0.010–0.247)^**^
*Oscillibacter*	3.914	0.006	1.042(0.263–1.317)	2.383(1.487–3.187)^*^	0.617(0.047–2.527)^#^
*Streptococcus*	3.350	0.013	0.135(0.013–0.913)	0.040(0.037–0.080)	0.223(0.150–2.067)^##^
*Lachnospiraceae_bacterium_COE1*	3.059	0.001	0.152(0.017–0.697)	0.003(0.000–0.010)^**^	0.003(0.000–0.023)^*^
*Christensenellaceae*	4.152	0.004	1.585(0.727–2.690)	4.122(2.450–6.210)^*^	1.280(0.077–2.093)^##^
*Prevotellaceae_NK3B31_group*	4.047	0.005	1.382(0.047–7.914)	0.067(0.003–0.113)	0.043(0.000–0.097)^**^
*Lactobacillus_animalis*	4.005	0.016	1.078(0.267–4.400)	0.212(0.030–0.683)^*^	0.913(0.413–10.697)^#^
*_Ruminococcus__gnavus*	3.653	0.001	0.073(0.007–0.213)	0.030(0.003–0.057)	0.370(0.180–2.793)^*^^,^^##^
*Acidaminococcaceae*	3.146	0.027	0.000(0.000–0.037)	0.000(0.000–0.017)	0.030(0.000–1.027)^#^
*Alphaproteobacteria*	3.101	0.005	0.033(0.020–0.080)	0.058(0.030–0.230)	0.117(0.083–1.333)^**^
*Rhodospirillaceae*	3.092	0.007	0.033(0.020–0.073)	0.053(0.030–0.230)	0.107(0.070–1.333)^**^
*Betaproteobacteria*	3.906	0.015	0.705(0.550–2.027)	1.723(1.293–4.280)	1.993(0.863–5.780)^*^
*Alloprevotella*	3.796	0.042	0.942(0.290–1.370)	1.942(0.160–3.433)	0.317(0.040–0.790)
*Negativicutes*	3.284	0.005	0.040(0.000–0.067)	0.013(0.000–0.033)	0.330(0.010–1.270)^##^
*Ruminococcaceae_UCG_013*	3.196	0.002	0.360(0.033–0.730)	0.090(0.037–0.110)	0.020(0.013–0.093)^**^
*Ruminococcaceae_UCG_010*	3.041	0.001	0.133(0.097–0.220)	0.317(0.220–0.470)^*^	0.097(0.030–0.190)^##^
*Desulfovibrio*	4.026	0.013	1.062(0.517–1.913)	2.417(1.797–5.130)^*^	0.847(0.557–5.667)^#^
*Deltaproteobacteria*	4.029	0.013	1.065(0.517–1.923)	2.433(1.827–5.147)^*^	0.853(0.557–5.707)^#^
*_Clostridium__leptum*	3.873	0.007	1.427(0.427–1.973)	2.610(1.653–4.987)^**^	1.607(0.423–4.903)
*Gammaproteobacteria*	3.827	0.018	0.203(0.043–0.380)	0.292(0.070–5.044)	1.357(0.277–3.113)^*^
*Lachnospiraceae_UCG_010*	4.023	0.004	0.335(0.067–0.843)	2.400(1.453–3.640)^**^	1.453(0.050–4.460)
*Phascolarctobacterium_faecium*	3.146	0.027	0.000(0.000–0.037)	0.000(0.000–0.017)	0.030(0.000–1.027)^#^
*Enterobacteriaceae*	3.826	0.016	0.187(0.043–0.353)	0.273(0.070–5.040)	1.357(0.263–3.087)^*^
*Family_XIII*	3.448	0.036	0.303(0.173–0.743)	0.903(0.520–1.293)^*^	0.580(0.110–1.710)
*Bacteroidales_S24_7_group*	4.883	0.007	22.127(16.074–31.254)	7.100(4.260–10.880)^*^	10.764(1.607–25.278)^*^
*Mollicutes*	3.180	0.040	0.217(0.060–0.350)	0.375(0.140–0.827)	0.103(0.007–0.293)^#^
*Lachnoclostridium*	3.803	0.003	0.205(0.083–0.333)	0.163(0.047–0.247)	0.493(0.237–3.730)^*^^,^^##^
*Peptococcaceae*	3.294	0.044	0.442(0.173–0.620)	0.673(0.310–1.420)	0.323(0.050–0.973)^#^
*Parasutterella*	3.904	0.015	0.697(0.543–2.013)	1.718(1.293–4.280)	1.983(0.853–5.760)^*^^,^^#^
*Prevotellaceae*	4.319	0.028	7.814(3.413–12.420)	4.167(0.893–11.080)	1.107(0.533–25.004)^*^
*Tenericutes*	3.180	0.040	0.217(0.060–0.350)	0.375(0.140–0.827)	0.103(0.007–0.293)^#^
*Ruminiclostridium_6*	3.869	0.010	0.387(0.137–0.917)	1.710(0.353–3.273)	0.210(0.027–1.687)^##^
*Ruminiclostridium_5*	3.101	0.005	0.598(0.513–0.747)	0.310(0.190–0.517)^*^	0.407(0.077–0.643)^*^
*Streptococcus_hyointestinalis*	3.350	0.015	0.125(0.010–0.907)	0.028(0.003–0.067)	0.207(0.137–2.040)^#^
*Christensenellaceae_R_7_group*	4.142	0.003	1.455(0.640–2.543)	4.008(2.323–6.034)^*^	1.203(0.070–2.010)^##^
*Family_XIII_AD3011_group*	3.075	0.037	0.092(0.047–0.257)	0.315(0.127–0.633)^*^	0.190(0.033–0.473)
*Selenomonadales*	3.284	0.005	0.040(0.000–0.067)	0.013(0.000–0.033)	0.330(0.010–1.270)^##^
*Proteobacteria*	4.370	0.003	2.180(1.260–3.910)	5.952(3.557–14.327)^**^	5.760(2.603–11.244)^*^
*Bacteroidaceae*	4.726	0.006	3.168(0.880–17.521)	7.917(3.180–14.854)	12.584(9.930–28.454)^**^
*Lachnospiraceae_ND3007_group*	3.423	0.001	0.048(0.020–3.950)	0.012(0.000–0.033)^*^	0.003(0.000–0.013)^**^
*Thalassospira*	3.092	0.007	0.033(0.020–0.073)	0.053(0.030–0.230)	0.107(0.070–1.333)^**^
*Rhodospirillales*	3.092	0.007	0.033(0.020–0.073)	0.053(0.030–0.230)	0.107(0.070–1.333)^**^
*Alcaligenaceae*	3.904	0.015	0.705(0.550–2.027)	1.723(1.293–4.280)	1.993(0.853–5.767)^*^
*Lachnospiraceae_UCG_005*	3.961	0.008	0.757(0.270–1.470)	2.562(1.710–2.987)^*^	3.050(0.130–4.503)^*^
*Desulfovibrionaceae*	4.029	0.013	1.065(0.517–1.923)	2.433(1.827–5.147)^*^	0.853(0.557–5.707)^#^
*Escherichia_coli*	3.817	0.035	0.160(0.040–0.297)	0.210(0.057–4.927)	1.237(0.147–2.987)^*^
*Bacteroides_acidifaciens*	4.030	0.002	0.247(0.103–1.103)	0.125(0.023–0.337)	1.610(0.540–6.377)^##^
*Burkholderiales*	3.906	0.015	0.705(0.550–2.027)	1.723(1.293–4.280)	1.993(0.863–5.780)^*^
*Anaerotruncus*	3.648	0.005	0.612(0.400–0.790)	1.618(0.997–1.980)^**^	0.820(0.160–1.633)

**Notes.**

Data are presented as median (minimum-maximum), *n* = 6 in each group. ^∗^*P* < 0.05, ^∗∗^*P* < 0.01, ^∗∗∗^*P* < 0.001 versus CON group, ^#^*P* < 0.05, ^##^*P* < 0.01 versus DM group (Kruskal–Wallis test and the Mann–Whitney test).

### Correlations between metabolic biomarkers and bacterial abundance

**Figure 8:**
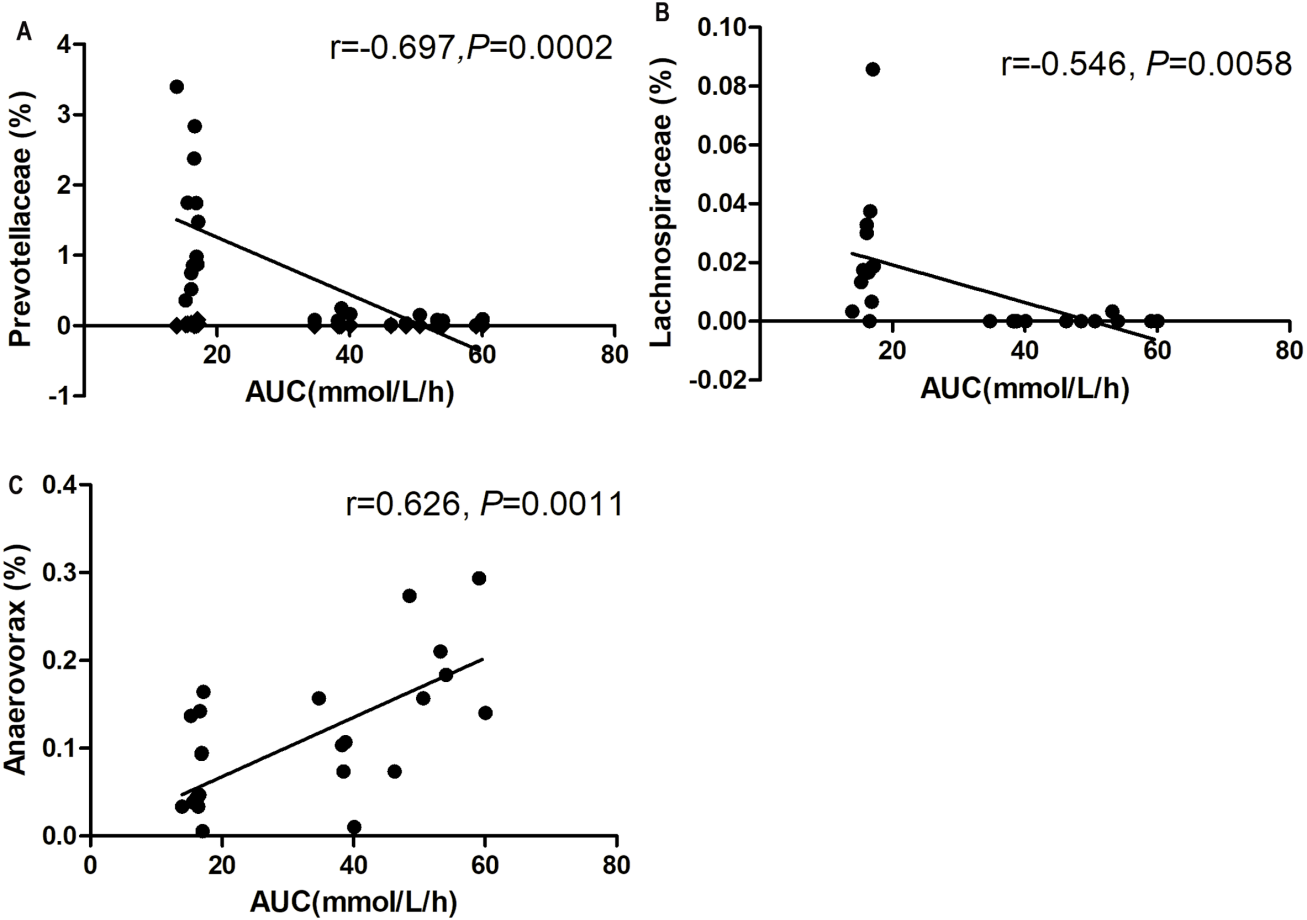
Correlation analysis between relative abundance (%) of gut bacteria and blood glucose response to glucose load. (A) *Prevotellaceae_UCG-001*, (B) *Lachnosporaceae_UCG-006*, and (C) *Anaerovorax*. *n* = 6 in each group (Spearman’s correlation analysis).

At the genus level, *Prevotellaceae_UCG-001* and *Lachnospiraceae_UCG-006* abundances were negatively correlated with blood glucose AUC on the OGTT (*P* < 0.01, Figs. 8A and 8B). The *Anaerovorax* abundance showed a positive correlation with blood glucose AUC on the OGTT (*P* < 0.01, Fig. 8C).

## Discussion

In this study, we employed a combination of high-fat diet and low-dose STZ injections to mimic human T2DM characteristics, including hyperglycemia, hyperinsulinemia, and dyslipidemia. In this protocol, low-dose STZ injections were administered to rats to slightly damage pancreatic β cells. Furthermore, long term feeding of the high-fat diet induced insulin resistance and dyslipidemia. This T2DM animal model has been used by several studies (Chen et al., 2017; Stalin et al., 2016). Interestingly, we found that inulin treatment effectively moderated glucose intolerance, insulin resistance, and blood lipid panels in the HF/STZ-induced diabetic rat model. A recent meta-analysis showed that inulin-type fructans reduce fasting blood glucose in T2D patients (Liu et al., 2017). Moreover, in pre-diabetic subjects, inulin moderated insulin resistance in an impaired fasting blood subgroup (Guess et al., 2016). A combined supplement of inulin and fiberol-2 (another type of soluble dietary fiber) induced in a reduction in blood lipids in hamsters with hyperlipidemia (Huang et al., 2016). Moreover, inulin treatment increased serum GLP-1 level, reduced serum IL-6 level and *Il6* expression in white adipose tissue of diabetic rats. Previous research indicated that GLP-1 agonist (exendin-4) could inhibit the secretion of IL-6 (Guo et al., 2016). GLP-1 inhibit macrophage inflammation both in the human cell and the animal model (Buldak et al., 2016; Lee et al., 2012). IL-6 has been proven to have effects in various tissues including liver (Adser et al., 2011; Banzet et al., 2009; Pedersen et al., 2011). It is reported that the injection of recombinant IL-6 in rats resulted the increase of hepatic *Il6* and *Pepck* expression (Banzet et al., 2009). In our research, HF/STZ-induced diabetic rats had increased expression of hepatic gluconeogenesis. Inulin treatment suppressed hepatic *Pepck* and *G6pc* expression. Thus, enhanced serum GLP-1 levels induced by inulin may be correlated with the reduction of IL-6 production and secretion and the suppression of hepatic gluconeogenesis, resulting in the moderation of insulin resistance in diabetic rats.

According to an alpha diversity analysis, we found that bacterial diversity and richness significantly decreased in the DM + inulin group compared to that in the DM group. An analysis of unweighted UniFrac confirmed the distinct clustering of the relative abundances of OTUs among CON, DM, and DM + inulin rats. However, in normal rats, inulin did not influent alpha and beta diversity of the gut microbiota. Therefore, inulin administration may reduce the whole microbiota diversity and shape the specific structure of the gut microbiota only in diabetic rats, not in normal rats.

Our data showed that the *Firmicutes*: *Bacteroidetes* ratio was increased in DM rat fecal microbiota. Inulin treatment reduced this ratio. Previous studies reported that there was a positive correlation between the *Firmicutes*: *Bacteroidetes* ratio and both obesity and diabetes (Ley et al., 2005; Ley et al., 2006). Dietary porphyrin (a water soluble fiber) prevented hyperglycemia and substantially enhanced *Bacteroides* abundance in the cecum of KKAy diabetic mice (Kitano et al., 2012).

Moreover, we found that inulin treatment increased *Lactobacillus* abundance in gut microbiota. As a major probiotic, *Lactobacillus* are considered to be key bacteria, that benefit the health of the intestinal tract (Nobaek et al., 2000). *Lactobacillus* produces lactic acid, CO_2_, acetic acid, and/or ethanol, which contribute to a more acidic environment through homo- or heterofermentative metabolism (Macfarlane & Macfarlane, 1997). Previous studies found that *Lactobacillus* decreased in diabetic rats (Yan et al., 2016) and HF rats (Lau et al., 2016). Inclusion of feruloylated oligosaccharides from maize bran in the normal diet of rats increased *Lactobacillus* in fecal microbiota (Ou et al., 2016). Whole wheat consumption was associated with a three-fold higher abundance of *Lactobacillus* compared to both obese and lean control mice (Garcia-Mazcorro et al., 2016). Inulin treatment selectively stimulated beneficial Lactobacilli *in vitro* (Scott et al., 2014; Van de Wiele et al., 2007) and in human subjects and rodents (Campbell, Fahey & Wolf, 1997; Ramirez-Farias et al., 2009; Weitkunat et al., 2015).

In addition, we found that at the genus level, inulin treatment increased the abundances of *Bacteroides, Phascolarctobacterium*, and *Lachnoclostridium*. *Lachnospiraceae* abundance was negatively correlated with AUC on the OGTT. *Bacteroides*, *Phascolarctobacterium*, and *Lachnospiraceae* are SCFA-producing bacteria. SCFAs have been recently demonstrated to be key regulators of host metabolism and immunity. SCFAs are produced by the gut microbiome. The main components of SCFAs are acetates, propionates and butyrates and are produced through anaerobic fermentation of carbohydrates from indigestible dietary fiber (Park et al., 2015). SCFAs are absorbed and utilized by colonocytes and peripheral tissue for energy or act as substrates for lipogenesis, gluconeogenesis or regulation of cholesterol synthesis in the liver. Knockdown of SCFA receptors leads to inflammation, glucose intolerance and diet-induced obesity in mice (Bellahcene et al., 2013). Butyrate supplementation prevented insulin resistance and obesity in mice (Gao et al., 2009). Recently, a randomized double-blind, placebo-controlled trial indicated that butyrate and insulin supplementation significantly reduced fasting blood glucose and waist to hip ratio in T2DM patients (Roshanravan et al., 2017). Previous studies showed that the abundance of *Bacteroides* in T2DM Chinese patients was only half that of normal glucose tolerance subjects and prediabetics (Zhang et al., 2013). Wu et al. (2010) also found that *Bacteroide vulgatus* was less represented in the microbiota of a diabetic group than in the microbiota of a non-diabetic group. Both berberine and metformin increased *Bacteroides* abundance in HF diet-induced obese rats (Zhang et al., 2015). Physically fit healthy subjects showed increased abundances of *Lachnospiraceae* in fecal microbiota and increased production of fecal butyrate. *Lachnospiraceae* abundance exhibited positive correlations with peak oxygen uptake, the gold standard measure of cardiorespiratory fitness (Estaki et al., 2016). Both berberine and metformin treatment markedly increased the abundance of *Phascolarctobacterium* in gut microbiota (Zhang et al., 2015).

Our data also showed that *Desulfovibrio* abundance was lower in inulin-treated rats than in DM rats. As Gram-negative bacteria, most members of *Desulfovibrio* are lipopolysaccharide (LPS) producers (Loubinoux et al., 2000; Weglarz et al., 2003) and damage the gut barrier (Beerens & Romond, 1977). HFD can induce a leaky gut and cause bacterial lysis, allowing the LPS of Gram-negative bacteria to enter the enterohepatic circulation (Chang et al., 2015). LPS can activate pro-inflammatory cytokine production, leading to impaired insulin sensitivity and induction of insulin resistance-related metabolic disorders (Cani et al., 2008). Our results also showed that inulin treatment reduced serum IL-6 levels and adipose tissue *Il6* expression in diabetic rats. Previous studies found that the family *Desulfovibrionaceae* caused extensive impaired glucose tolerance (IGT)/obese rats (Zhang et al., 2010). Consumption of the Western diet (high fat, high sugar) for one month led to increased LPS in healthy individuals (Pendyala, Walker & Holt, 2012). Xie et al. (2016) found that *Desulfovibrio* abundance was markedly increased in STZ-HFD induced nonalcoholic steatohepatitis and was positively correlated with LPS levels.

*Ruminococcaceae* abundance was lower in inulin-treated rats than in non-treated diabetic rats. In another study, there was a reduction in the abundance of *Ruminococcaceae* in bitter melon formulation-treated rats (which reduced fasting blood glucose) compared to diabetic rats (Zhu et al., 2016). *Oscillibacter* is one species within the *Ruminococcaceae* family whose abundance is positively correlated with gut permeability, which can affect gut barrier integrity (Lam et al., 2012). The abundance of *Oscillibacter* increased under HFD conditions, and the abundance of *Oscillibacter* declined after inhibition of the mTOR complex (Jung et al., 2016).

## Conclusion

In summary, our study supports the hypothesis that inulin-induced changes to the composition of the gut microbiota in diabetic rats are linked to the anti-diabetic effects of inulin. In particular, inulin treatment enhanced the abundance of beneficial bacteria, including SCFA-producing bacteria and probiotic bacteria, and reduced the abundance of LPS-producing bacteria in the gut. Moreover, inulin treatment enhanced serum GLP-1 level to suppress IL-6 secretion and production and hepatic gluconeogenesis, and resulted in moderation of insulin tolerance. These findings indicate that gut-liver crosstalk is the main mechanism in moderation of insulin resistance by inulin. Our study provides evidence that the gut microbiota may be a relevant diabetes treatment. More studies are needed to investigate whether or not inulin treatment can directly moderate gut microbiota and glucose metabolism.

##  Supplemental Information

10.7717/peerj.4446/supp-1 Data S1 Raw data

## Additional Information and Declarations

### Data Availability

The following information was supplied regarding data availability: The raw data is provided in Data S1.
